# Unravelling the Drug Encapsulation Ability of Functional DNA Origami Nanostructures: Current Understanding and Future Prospects on Targeted Drug Delivery

**DOI:** 10.3390/polym15081850

**Published:** 2023-04-12

**Authors:** Souvik Ghosal, Sagar Bag, Sudipta Bhowmik

**Affiliations:** 1 Mahatma Gandhi Medical Advanced Research Institute (MGMARI), Sri Balaji Vidyapeeth (Deemed to Be University), Pondy-Cuddalore Main Road, Pillayarkuppam, Pondicherry 607402, India; 2 Department of Biophysics, Molecular Biology and Bioinformatics, University of Calcutta, 92, A.P.C. Road, Kolkata 700009, India

**Keywords:** DNA origami, DNA nanostructures, self-assembly, drug encapsulation, targeted drug delivery, disease therapeutics

## Abstract

Rapid breakthroughs in nucleic acid nanotechnology have always driven the creation of nano-assemblies with programmable design, potent functionality, good biocompatibility, and remarkable biosafety during the last few decades. Researchers are constantly looking for more powerful techniques that provide enhanced accuracy with greater resolution. The self-assembly of rationally designed nanostructures is now possible because of bottom-up structural nucleic acid (DNA and RNA) nanotechnology, notably DNA origami. Because DNA origami nanostructures can be organized precisely with nanoscale accuracy, they serve as a solid foundation for the exact arrangement of other functional materials for use in a number of applications in structural biology, biophysics, renewable energy, photonics, electronics, medicine, etc. DNA origami facilitates the creation of next-generation drug vectors to help in the solving of the rising demand on disease detection and therapy, as well as other biomedicine-related strategies in the real world. These DNA nanostructures, generated using Watson–Crick base pairing, exhibit a wide variety of properties, including great adaptability, precise programmability, and exceptionally low cytotoxicity in vitro and in vivo. This paper summarizes the synthesis of DNA origami and the drug encapsulation ability of functionalized DNA origami nanostructures. Finally, the remaining obstacles and prospects for DNA origami nanostructures in biomedical sciences are also highlighted.

## 1. Introduction

Naked therapeutic materials that include small and biomolecular drugs have several inherent difficulties that impede them from executing their activities fully in the body. This includes lower solubility, as well as stability against chemical and enzymatic breakdown, difficulty to cross biologic barriers, undesired side effects, and toxicity [1,2,3]. Several alternative drug delivery approaches have been developed throughout the decades to address these concerns [4]. Drug delivery carriers have emerged as a significant tool in current pharmaceutics, with the potential to reduce toxicity, enhance solubility, and improve targeting ability [3,4]. An effective drug delivery carrier should be non-toxic, simple to load drugs with biological functions, and capable of controlling drug release efficiencies [2]. DNA is one of the essential compounds found in almost all living things. Beyond biological significance, the last three decades have witnessed a significant advancement in the field of “DNA nanotechnology”, which uses DNA as a building block to create materials at the nanoscale [2,3]. The benefits of using DNA as a building material include: (A) Consistent and predictable structural parameters (double-stranded DNA has a diameter of 2 nm and a persistence length of 50 nm); (B) Highly conserved hydrogen bonding between nucleobases (A bonds with T and G bonds with C), which results in a completely predictable interaction and the formation of branched DNA motifs; and (C) Low-cost (bio) chemical synthesis. The tremendous flexibility of DNA structures allows for the modification of electrical characteristics through the use of external fields. Moreover, required structures may be produced at room temperature in an environmentally friendly and toxicity-free manner. The semiconducting capabilities of DNA-based devices have been facilitated by external electric and magnetic fields. Many applications that utilize DNA structures including nanomachines, nano-electronic materials were revealed previously [5]. Drug delivery methods based on nanoparticles are being employed in a wide range of applications. Some of the greatest possibilities for drug delivery systems are zinc oxide nanoparticles with high thermal stability and biocompatibility, excellent biological characteristics, and high selectivity [6]. In a study it was found that the presence of iron as an impurity in the zinc oxide nanostructure improves the power oxidation of zinc oxide nanostructures, leading to enhanced antibacterial activity [7].

To combat numerous illnesses, many nucleic acid therapies and chemotherapeutic drugs have been designed. Nowadays, synthetic drug deliveries are problematic because of these current limiting factors, such as stability, targeted transportation, solubility, regulated release, systemic distribution, and membrane penetration. Considering the potential immunogenicity and cytotoxicity of such carriers, their broad applicability is restricted. Through nucleic acid alterations, flexible chemically modified DNA origami nanostructures can be constructed. Because of its rich responsiveness and programmable sequences, DNA has gained increasing interest in the development of nanomaterials with predictable nanostructures and customizable functionalities, which have shown significant prospects in drug delivery. On the one hand, through sequence designing, DNA sequences with responsiveness, molecular recognition, and therapeutic effectiveness may be readily incorporated into the framework of DNA nanostructures. Drug delivery is a prospective application for these chemically altered DNA origami nanostructures with their increased stability and connected functional moieties via chemical alterations. DNA origami can be used as a drug loading vehicle to treat several diseases, such as multidrug-resistant leukemia, breast cancer, etc. [8]. The drugs that can be encapsulated in DNA origami are anthracycline doxorubicin, epirubicin, anthracycline daunorubicin, BMEPC, 56MESS, Aclarubicin, idarubicin, luteolin, and actinomycin-D [9,10]. DNA origami structures offer enormous potential for several applications, including nanofabrication, nanoplasmonics, nanoelectronics, catalysis, (bio) sensing, drug/gene delivery, and bioimaging, because of their inherent biocompatibility, simplicity in production, and ability to be chemically modified at precise sites [11]. Developing innovative cancer theragnostic, or a combination of cancer therapeutic and diagnostic agents, is an exciting example of one of the important applications [12]. These delivery methods may substantially enhance drug loading efficiency, circulation duration in the body, and final therapeutic effects through optimal parameter design. Several of these have undergone clinical studies and some have been permitted for clinical use.

DNA-based nanotechnology and, in particular, the DNA origami technique are progressively approaching real-world biological applications. Unfortunately, many of these applications are still restricted by the limited stability of DNA nanostructures in biological fluids. The presence of digestive enzymes and the low magnesium ion concentration of the DNA nanostructures might cause unwinding and structural collapse [13], limiting their lifespan. As a result, initiatives have been taken to protect DNA nanostructures from their surroundings, such as encapsulation, transferring their structural details into other materials, the chemical or enzymatic ligation of the staple strands, or covalently cross-linking neighboring DNA domains. Therefore, DNA origami design choices such as lattice type, staple lengths, crossover position and spacing, twist corrections, and so on all impact the mechanical and structural characteristics, and consequently, the environment-dependent behavior of the nanostructures [14].

This review will address the uses of DNA origami nanostructures in precision nanomedicine and evaluate their utility and practicality. The discussion will begin with an overview of DNA origami and some of the structures that may be constructed. After that, an explanation of the synthesis of DNA origami, the mechanism utilized to make DNA origami nanostructures, and recent applications of this technology for the targeted drug delivery will be provided. We will also discuss our prospective on the future of DNA-origami-based drug carriers. We believe that this review will not only rigorously explain current advancements in DNA origami, but will also inspire both scientists and beginners to promote the improvements in this fascinating area.

## 2. Overview and Structural Features of DNA Origami Nanostructures

The area of DNA nanotechnology has experienced a revolution with the introduction of DNA origami technology, which involves folding long viral “scaffold DNA strands” with chemically manufactured “staple DNA strands” into particular 2D and 3D structures [4]. These benefits enable the bulk design and fabrication of nanostructures with a predefined size, shape, and complexity in a very short amount of time. Many common strands are arranged in unusual ways in DNA origami. Staple strands are altered using well-known conjugation chemistry to provide targeting, imaging, and therapeutic modalities [4,5].

DNA origami technology is a valuable technique for building well-defined nanostructures from the bottom up, with sizes ranging from tens of nanometers to sub-micrometers. It is a promising field of DNA nanotechnology. DNA is folded at the nanoscale to create 2D and 3D objects in DNA origami. Hundreds of specially made small single-stranded DNAs called staples are utilized to fold a large single-stranded DNA known as the scaffold, which is generally viral DNA and is around 7000 nucleotides long. Each staple includes a number of binding domains that, by crossover base pairing, connect and bind the previously dispersed parts of the scaffold, folding it similarly to knitting [1]. The staple sequences can be used to program the geometry of the resultant structures. Because DNA origami is programmable, computer-aided design and universal synthesis procedures are possible, making it a simple technology that can be produced automatically [2,4]. As compared to tile-based DNA assembly techniques, DNA origami synthesis frequently exhibits greater yield, durability, and the ability to create intricate non-periodic structures. This is mainly due to the strong cooperativity of many scaffold-staple connections during origami folding [5,6]. Since the initial display of 2D patterns [1], it is now possible to create almost any arbitrary shape, including 1D and 3D structures with user-defined asymmetry [7,8], cavities, or curves [9,15]. The dynamic structures, single-stranded origami, and hierarchical assembly of supramolecular structures are examples of more recent advancements [16,17,18]. A typical planar DNA origami construction comprises 200 staples with different sequences and locations that can serve as uniquely addressable sites over an area of 8000–10,000 nm^2^ [2]. By prescribing functional moieties on staples, various forms of material may be site-specifically inserted at designated spots on a DNA origami structure, allowing the structures to behave as sophisticated pegboards or frameworks [19,20,21]. Dynamic DNA origami structures can be rationally engineered on the basis of structurally reconfigurable modules that use conformationally switchable domains, strand displacement reactions, and base stacking components. This enables a variety of applications, including smart drug delivery, target-responsive biosensing and bioimaging, nanodevices and biomolecular computing that can be externally manipulated with light or other electromagnetic fields [22,23,24].

## 3. Synthesis and Assembly of DNA Origami Nanostructures

**Figure 1:**
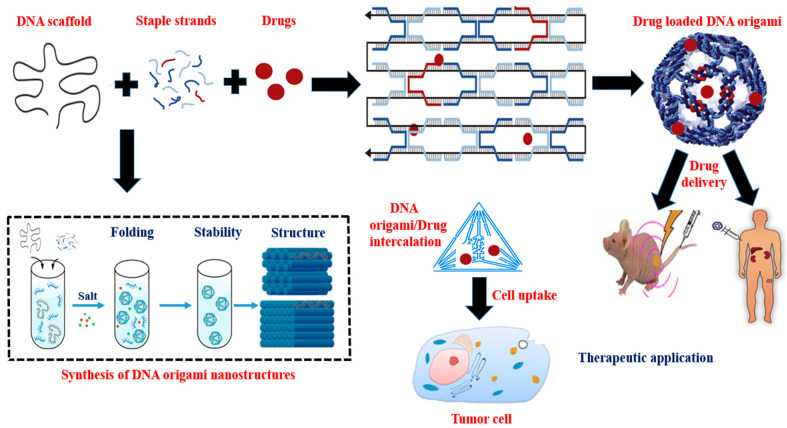
Structural formation and therapeutic application of DNA origami nanostructures. The complimentary staple strands are used to fold a long single-stranded DNA scaffold. Cargo is added via direct conjugation, encapsulation, intercalation, and hybridization of a single strand DNA-functionalized drug onto an extended staple strand. DNA origami nanostructure has immense potential as an efficient drug carrier and delivery vehicle in cancer treatment.

Paul Rothemund created one of the most important developments in DNA nano-construction [25]. He described a fundamentally novel method for creating discrete DNA nanostructures that uses several short DNA “staple” strands to control how a long “scaffold” strand folds into a flat array of antiparallel helices (Figure 1). “Scaffolded DNA Origami” provides a number of significant benefits over conventional assembly methods. A lengthy strand of DNA is folded without many mistakes, producing final structures with fewer flaws and a greater yield. The process reduces stoichiometric dependency, obviates the need to purify the oligonucleotides, and shortens the synthesis time. More sophisticated forms may be produced, and the resultant nanostructures, which have defined dimensions and are entirely programmable, enable the attachment of molecules at specific locations. Because staples must bind to the scaffold and not to one another, the relative concentrations of the staples are not important for the effectiveness of scaffolded DNA origami. Accurate staples are first attached, partially arranging the lengthy scaffold for the correct binding of the remaining staples. The correct binding of staples can then displace wrong or truncated staples and remove undesirable secondary structure thanks to strand invasion. Since Rothemund’s first study, several research teams have built a variety of structures using scaffolded DNA origami and demonstrated a number of significant applications [26]. In previous research, the circular, single-stranded M13mp18 genome was folded into an antiparallel array of helices using a pattern of periodic cross-overs using 200 staple strands. Astonishingly, the high yields of the desired structure are produced by the self-assembly process, which involves annealing the template for roughly an hour in the presence of typically 100-fold too many staple strands. Given that the multistranded assembly of DNA oligonucleotides into extended superlattices typically requires up to 20 h, the speed of the thermal annealing (from roughly 95 °C to room temperature) is noteworthy (Figure 1). The DNA origami method’s remarkable performance is mostly owed to the entropic benefit of having a single long scaffold strand for folding [26,27].

Three-dimensional DNA origamis constructed on a lattice assist to a better knowledge of physical processes such as protein associations, plasmonics, single-molecule force studies, and enzymatic cascades reactions. Furthermore, several investigations suggested that by strand displacement, 3-dimensional DNA origamis are applicable for drug delivery vehicles, nano-devices, nano-switches. The advent of a user-friendly software program, caDNAno, facilitating structure design and permitting non-specialists from other areas to participate in 3D DNA origami design in a short period has tremendously aided the performance of scaffold-based lattice-engineered 3-dimensional DNA origami. Three-dimensional DNA origami was used to simulate channel proteins, which allow for transport across membrane lipid layers, cellular absorption of drug-loaded nanostructured materials, and systemic administration in vivo. Briefly, 3-dimensional DNA origami was studied as a prospective bottom-up upgrade to solid-state nanopores, which are generally organized using top-down lithography and etching methodologies, in order to provide advanced functions, for example, by inserting a funnel-shaped origami into the solid-state nanopores in a silicon-nitride membrane. In a new direction, 3D lattice-engineered DNA origami efficiently targets and delivers the well-known anticancer drug carboplatin to uterine cancer cells [28]. The 3D lattice-engineered DNA origami structure will be more effective in targeting and eliminating specific receptor-associated overexpression cells than nontargeted origami. This discovery, in our opinion, will enable the targeted delivery of anticancer drug combos to drug-resistant cancer cells utilizing adaptable DNA origami nanostructures. The experimental production of 3D DNA origami includes mixing all strands, often using a 2–10× excess of the staple strands, adjusting Na^+^ and Mg^2+^ levels, and renaturation via a gradually declining temperature ramp beginning at higher temperatures. Some techniques, however, employ isothermal foldable methods based on various denaturation procedures [29]. Excess staples are removed from the folded assemblies using rate-zonal ultracentrifugation, gel electrophoresis, spin-filtration, and size-exclusion chromatography. The method of purification used is determined by the structure’s architecture, as well as the magnitude and needs of future applications. Several scaffold logic gates, such as XOR and AND, may be developed using DNA tetrahedral nanostructures. A variety of programmable DNA tetrahedron nanostructures featuring dynamic sequences sensitive to tiny molecules (ATP), protons, metal ions (Hg^2+^), and complementary nucleic acid strands (T7 RNA transcription and miRNA) have been constructed. These DNA-nanostructure-based logical computations can identify disease biomarkers while also controlling the in vivo release of tiny chemicals. A multipurpose optical sensing platform based on DNA-tetrahedron-linked hairpin probes may be built for numerous investigations of endonucleases, small compounds, and miRNAs [30].

## 4. DNA-Origami-Based Approaches and Therapeutic Strategies for Targeted Drug Delivery

Due of its full addressability and greater yields, DNA origami provides a superb platform for organizing matter with the greatest accuracy and control. Below, we discuss several compounds and the strategy they used for targeted drug delivery. Doxorubicin, epirubicin, daunorubicin, aclarubicin mitoxantrone, and cisplatin are chemotherapeutic drugs. Six-bis[2-(1-methylpyridinium)ethynyl]-9-Pentylcarbazole Diiodide (BMEPC) is a carbazole-derived photosensitizer. 56MESS is a platinum-based compound. Quercetin and luteolin belong to plant flavonoids. Actinomycin-D is a natural chromopeptide.

**Figure 2:**
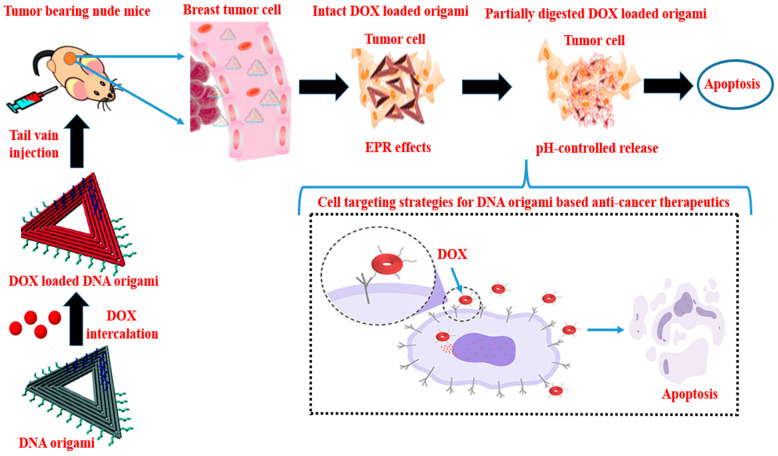
DOX loaded distribution of triangular origami and pH-controlled release in vivo, revealing strong passive DOX accumulation in cancerous regions and overall excellent therapeutic benefits in vivo.

After assembling hundreds of complementary DNA helper strands and M13mp18 phage DNA into DNA origami carriers, the anthracycline doxorubicin (DOX) drug was noncovalently intercalated into the carriers. A thorough analysis of the anticancer effects of DOX/origami in vivo was carried out [27] (Figure 2).

During a continuous 12-day course of treatment, tumor-bearing mice with tumor volumes of around 100 mm^3^ were divided into four groups (control, DOX, DOX/origami, and origami). DOX and DOX/origami intravenous injections at dosages equivalent to 4 mg/kg of DOX each were administered every three days. As a control, 0.9% saline was administered in the same volume as bare origami (0.08 mg/kg/day). When compared to the saline-treated group, the nude mice treated with DOX and DOX/origami both showed tumor volume suppression effects. In addition, the DOX/origami therapy showed a considerably greater ratio of lowering tumor burden than the DOX-treated group. Using the empty DNA origami, there was no tumor inhibition. In summary, DOX containing DNA origami that was directed on a breast tumor in BALB/c mice showed excellent anticancer activity without any systemic harm [27,31]. 

In a recent investigation, it has been found that all DNA origami nanostructures (DON) have equivalent DOX-binding capabilities (one DOX molecule for every two to three base pairs), and the binding equilibrium is achieved in seconds, which is much faster than previously anticipated. The degradation of DON and DOX releases from complexes during the digestion of DNase I were examined to describe drug release characteristics. The proportional dosages (DOX molecules released per unit time) of the utilized DONs might differ by two orders of magnitude depending on the DON structure. Moreover, from this study, the DOX aggregation processes, as well as spectrum alterations associated with pH, magnesium, and DOX concentration have been uncovered [32]. 

**Figure 3:**
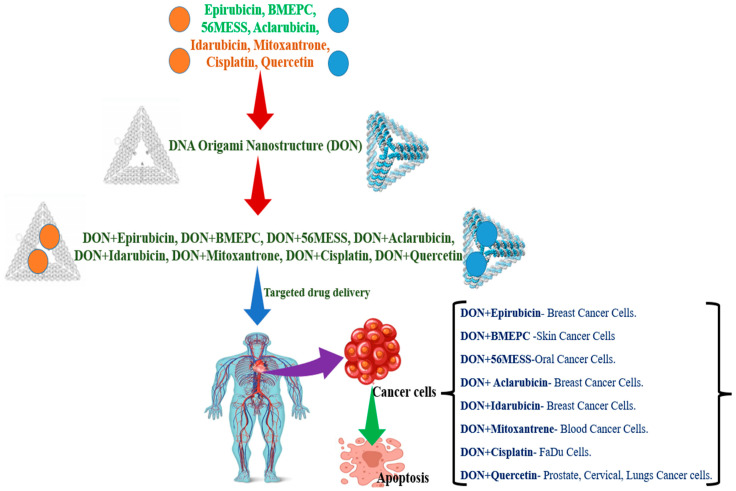
Schematic representation of DNA-origami-nanostructure-mediated targeted drug delivery for cancer therapy. [Epirubicin, BMEPC, 56MESS, Aclarubicin, Idarubicin, Mitoxantrone, Cisplatin, Quercetin are drugs; DON-DNA origami nanostructure].

Epirubicin is a planar anthraquinone nucleus connected to an amino-containing sugar that functions as an antitumor antibiotic. The semi-synthetic equivalent of DOX, epirubicin, which is intercalated non-covalently into the double-stranded DNA 5′-CG- 3′ and the 5′-GC-3′ in DNA origami and differs from it only in terms of stereochemistry, is similarly effective in treating breast cancer (Figure 3).

Trojan “Horse” DNA origami nanostructures in the form of rods were used to transport the anthracycline daunorubicin to multidrug resistant HL-60/ADR human leukemia cells. It has been discovered that daunorubicin-loaded Equine DNA nanostructures may overcome multidrug resistance protein-1 mediated drug resistance in a leukemia model [33]. 

Six-bis[2-(1-methylpyridinium)ethynyl]-9-Pentylcarbazole Diiodide (BMEPC) is a component of photo dynamic treatment (PDT), a cancer therapy option that uses ultraviolet or visible light in combination with a photosensitizer and molecule oxygen. This mixture has the potential to create extremely reactive oxygen species, which may eventually result in the multiple processes that destroy tumor cells. BMEPC may be added to DNA origami, which tumor cells can absorb. When exposed to radiation, BMEPC can produce free radicals and cause apoptosis (Figure 3). This finding indicates that BMEPC-loaded DNA origami complexes have superior imaging and photodynamic capabilities than carrier-free BMEPCs, making them an attractive choice for intracellular imaging and cancer treatment [34].

In order to accomplish a multidrug combination therapy, a nano antibody that targets the inhibition of the EGFR (epidermal growth factor receptor), one of the tumor indicators, was mixed with the platinum-based medication 56MESS and intercalated into a tetrahedron DNA origami. It has been discovered that the DNA nanoplatform and nanobodies worked together to treat tumors with exceptional selectivity and without any discernible harm [35].

Aclarubicin is an anti-cancer drug used to treat acute nonlymphocytic leukemia intercalated into DNA origami [36]. When first-line chemotherapy fails to treat advanced breast cancer, idarubicin is administered. [36,37].

Mitoxantrone is a drug that is loaded into the double strands of the folate-overhung tetrahedron DNA origami and utilized as an anticancer agent. The folate-overhung mitoxantrone tetrahedra DNA origami (about 25 nm) may target leukemic cells, travel through the nucleus membrane, trigger apoptosis, and improve the overall effectiveness of treating leukemic cells in vitro and in mice with leukemia (Figure 3) [38].

Drug cisplatin is used to assess the cytotoxicity of DNA origami nanostructures on FaDu cells, as well as to cross-link the structures (a punch biopsy of a hypopharyngeal tumor taken from a 56-year-old white male patient with squamous cell carcinoma resulted in the establishment of the cell line FaDu, which has epithelial morphology). After 48–72 h, cell viability from nanomolar doses of cisplatin-loaded DONs is reduced to 50% (Figure 3) [39].

The anti-inflammatory, antioxidant, toxic, anti-cancer, and immunomodulatory properties of quercetin (flavonoids) demonstrate its potential therapeutic value. Despite its many positive effects on human health, quercetin has some drawbacks, including its hydrophobic nature, low bioavailability, poor solubility, and poor permeability. Quercetin is encapsulated in DNA origami to improve its solubility and absorption in order to overcome some of its drawbacks [40]. A typical flavonoid is luteolin, a 3′,4′,5,7-tetrahydroxyflavone that may be found in a wide range of plants, including fruits, vegetables, and medicinal herbs. Luteolin-rich plants have been utilized in Chinese traditional medicine to treat a range of diseases, including cancer, inflammatory conditions, and hypertension. Luteolin contains a range of biological effects, such as anti-allergy, anti-inflammation, and anticancer (Figure 3). It may function biochemically as either an antioxidant or a pro-oxidant. The biological effects of luteolin could be related functionally. For instance, its anti-inflammatory activity could be connected to its anti-cancer effect. The stimulation of apoptosis and the prevention of cell growth, metastasis, and angiogenesis are two of luteolin’s anticancer properties. Other drawbacks include its hydrophobic nature, limited bioavailability, poor permeability, and poor solubility. Luteolin is encapsulated in DNA origami to improve its bioavailability and solubility in order to overcome some of its drawbacks [41].

The well-known antibiotic actinomycin-D, which is intercalated in DNA origami and has significant antibacterial and anticancer action, belongs to the actinomycin group. Its cytotoxic and antitumor effects are caused by a number of mechanisms, many of which are linked to DNA functioning, which inhibits the production of RNA and, in turn, protein synthesis. The two main mechanisms are intercalation to DNA and stabilization of topoisomerases I and II cleavable complexes with DNA, in which a polypeptide lactone ring occupies a position in the minor groove of the DNA helix, and a phenoxazone ring localizes between the GpC base pair sequence in DNA, or the drug penetrates to a location in the DNA structure where topoisomerase binds with DNA, respectively. In addition, it has been hypothesized that actinomycin D’s sluggish dissociation from DNA complexes, photodynamic activity, free radical generation, and other biochemical impacts of activity may be significant determinants of this drug’s biological action [42].

Since CpG patterns are much more common in microbial DNA than in vertebrate genomes, the immune system recognizes them as an indication of pathogen invasion. These sequences are identified by Toll-like receptor 9 (TLR9) when they are unmethylated, which can severely activate the innate and adaptive immune systems. As a result, CpG oligodeoxynucleotides are a strong option for use as an adjuvant in immunotherapy vaccines. The fact that native CpG dinucleotides are very vulnerable to nuclease degradation poses a problem for their application, hence stabilizing modifications, such as phosphorothioate (PTO)-modified backbones, are investigated. Due to the fact that PTO-CpGs are not as effective as adjuvants and may harm organs or lymphoid tissue, DNA origami is being investigated as a possible nanocarrier of CpG sequences for immunotherapies [43,44]. By hybridizing up to 62 distinct CpG sequences to staple tethers on the inner or outer surface of a 30-helix DNA origami tube, Schüller et al. investigated the application of DNA origami as a CpG nanocarrier [34]. The CpG sequences put to the test were all PTO-backbone changed, some of them only partially. These CpG-sequence-coated DNA origami tubes outperformed conventional carrier systems in inducing a strong immunological response when incubated with newly separated spleen cells through the pathway (e.g., Lipofectamine) [44]. In contrast to Lipofectamine, the origami carriers had no discernible cytotoxicity and had no effect on cell survival. Interestingly, whether the CpG sequences covered the inner or outer half of the origami tube had no influence on the efficiency of the origami nanocarriers, which is in line with the theory that the tubes deconstruct intra-endosomally. The partially PTO-backbone modified CpG sequences that were attached to the DNA-origami-based tubes produced the best reaction [45]. Nucleic acids with immunostimulatory and immunomodulatory properties are often used adjuvants in the immunotherapy of many illnesses [45]. A powerful systemic immunological response may be elicited by CpG by interacting with a variety of TLR. They can also be used as ingredients in immunotherapy vaccinations. Thrombosis, arthritis, lupus, and psoriasis have all been treated with these immunomodulatory nucleic acids [46]. Oligodeoxynucleotides (ODN) containing an unmethylated CpG pattern are regarded to be excellent immunotherapeutic vaccine adjuvants to help achieve successful therapeutic applications because they may stimulate TLR9. CpG has been investigated by clinical trial teams for glioblastoma multiforme, metastatic breast cancer, and melanoma immunotherapy. TLR9 activates immune-relevant cells, such as dendritic cells (DCs), macrophages, and B cells, to produce cytokines that promote inflammation. The DNA nanostructure is widely used to transport CpG, as seen in tubular DNA origami [47]. These pro-inflammatory cytokines are taken up by cells, identified by TLR9, and then produced to provide immunotherapeutic effects on a variety of disorders. The tumor necrosis factor (TNF), interleukin-6 (IL-6), interleukin-12 (IL-12), and co-stimulatory factors, including CD80 and CD86, are proinflammatory cytokines that are produced when TLR9 is bound [48]. Events such as these support APC survival and growth while encouraging Th1 immunostimulatory responses and inhibiting Th2 adaptive immunological responses [49].

In a recent study, the “Square block” DNA origami platform was introduced to investigate the significance of the spacing of CpG oligonucleotides that bind Toll-like receptors and so serve as danger signals for dendritic cells. It has been demonstrated that when CpG is separated at 3.5 nm, square blocks promote Th1 immune polarization in vivo and in vitro tumor-treatment models. This DNA origami vaccine improves DC activation, NK cell activation, antigen cross-presentation, Th1-polarized CD4 activation, and CD8 T cell activation. This research introduced a DNA-origami-based cancer vaccine that delivers antigen and CpG immune adjuvant with appropriate spacing for Th1 immune polarization [50].

Octahedral DNA origami used as a nano-vehicle for the delivery of siRNAs. Although photothermal treatment and chemotherapy are frequently employed to treat cancer, their effectiveness is frequently constrained by multidrug resistance. Small interfering RNAs (siRNAs) have been widely used in cancer therapy to battle multidrug resistance to chemotherapeutic medicines and hyperthermia because of their capacity to decrease the expression of target genes. It is still extremely difficult to administer siRNAs and chemo-photothermal agents effectively in vivo. In this study, octahedral DNA origami frameworks (OctDOFs) are built as a nano-vehicle for precisely organizing and orchestrating the distribution of siRNAs in combinational cancer treatment. In order to effectively downregulate the connective tissue growth factor (CTGF) and heat shock protein 72 (HSP72) for the dual sensitization of cancer cells to chemotherapeutic medicines and hyperthermia, the stiff OctDOFs structure’s inner cavity sterically prevents RNase destruction and protein binding. The suggested OctDOFs demonstrated improved cytotoxicity and tumor suppression efficacy in vitro and in vivo by increasing chemo-photothermal therapeutic potency with siRNAs. A novel siRNA delivery platform for targeted medicine and combination treatment is created by this nano-vehicle [51]. In a study, Church’s team put antibodies into a DNA origami barrel nanostructure, which used aptamer-based “locks” to regulate the transition between open and closed states. The barrel was opened to release Fab antibody fragments that bind to human CD33 and CDw328 to suppress the development of leukemia cells upon the identification of cell-surface receptor “keys” on the cell membrane [52]. The transport of gold nanorods (AuNRs) to the tumor site has been aided by the passive tumor-targeting ability of DNA origami, resulting in a more effective increase in local temperatures following near-infrared (NIR) irradiation and a greater photo thermal therapy effectiveness than AuNR alone [53,54]. These therapeutic systems’ ability to include imaging agents allowed for the real-time observation of their biodistribution and tumor uptake using fluorescence or optoacoustic imaging [55].

## 5. Cellular Targeting and Entry of Drug Encapsulated DNA Origami

**Figure 4:**
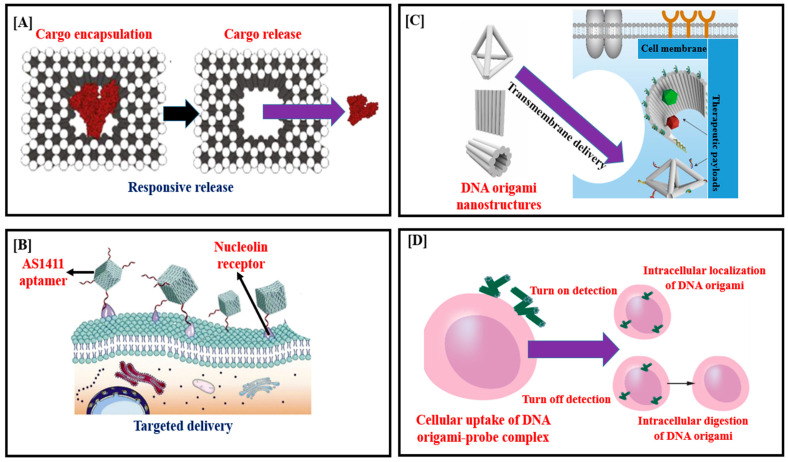
(**A**) The application of light-cleavable linkers allows for the responsive release of drugs from DNA origami nanostructures. (**B**) DNA origami can be delivered to nucleolin receptor-expressing tumor cells using nucleolin receptor-specific aptamers. (**C**) Biological function regulation by DNA origami nanostructures at cell interface. (**D**) Following the formation of the DNAorigami–probe complex, DNA origami can be delivered to the cells, and its intracellular location and breakdown can be detected using fluorescence microscopy.

Drug carriers must contain sufficient amounts of drug, which must be loaded and delivered in a regulated way. The cargo integration approach is determined by the drug’s characteristics, the kind of DNA origami, and the necessity to preserve drug activity after release. With continued advances in mass production and diverse ways for stabilizing DONs in physiological fluids, the possibility of DNA origami nanostructures for biological applications is being properly explored [8,56]. Although DNA origami nanostructures are often stiff, their durability in the cellular microenvironment is a critical obstacle for drug delivery. A potent method for delivering medicinal drugs is DNA origami [57] (Figure 4A–D). To target certain cell types, DNA origami enables the inclusion of functional groups, such as ligands (for example, folate), aptamers, or antibodies (Table 1).

The endosomal compartment is where drug-encapsulated DNA origami is typically held after absorption, where it must either escape or release a loaded payload. Using electroporation, which involves rupturing the outer membrane with a powerful electrical field to let the origami structures pass through, it is possible to directly transport drug-encapsulated DNA origami into the cytoplasm [44,56,64,65,66,67,68]. The use of DNA origami structures has been shown in several studies to provide control over the dosage of loaded medicine and to promote the cellular internalization of drug molecules that may ordinarily struggle to accumulate in cells (Table 1) (Figure 4A,B). By adopting sophisticated triggered-release mechanisms, the drug carrier origami may reversibly open and close, releasing payloads such as therapeutic proteins [69]. For instance, when using antibody fragments, such as the payload, S. Douglas et al. effectively administered pharmaceuticals using DNA nanorobots capable of functioning logic gates, molecular recognition and sensing, and smart payload release [51]. J. R. Burns et al. reported a DNA origami cube that uses non-covalently coupled HIV-Tat transduction domains and a reversible open and close lid to detect target sites and release protein in a paper that is identical to this one [70]. One of the most thoroughly investigated medical uses of DNA origami structures is the delivery of DNA intercalator drugs, such as DOX and daunorubicin. Moreover, enzymes [71], therapeutic proteins [72], and groove-binding medicines [73] all benefit from the use of DNA origami structures as delivery systems. In order to conveniently control the loading and release rates of DOX, Y. Zhao et al. created DNA origami nanotubes with various levels of global twist and structural relaxation, when used on three breast cancer cell lines, the more tightly twisted DNA nanotube design displayed stronger cytotoxicity and a lower rate of intracellular clearance than free DOX [74]. In a related study, DOX was loaded onto triangle- and tube-shaped DNA origami nanostructures with a loading efficiency of 60–70%. Both designs demonstrated noticeably higher rates of drug resistance cell death because of a higher DOX internalization when compared to free DOX and DOX-loaded double-strand M13 DNA. Moreover, DOX may be redistributed and lysosomal acidification might be suppressed by the DNA origami structures [75]. S. Palazzolo et al. developed 30 nm long by 10 nm broad compact short tube DNA origami constructs as an alternate method for DOX administration. These structures were enclosed in stealth liposomes, which allowed them to efficiently load DOX into the liposomes without the need for pH-driven gradients. They were very stable in physiological circumstances for more than 48 h [62]. Daunorubicin, an anthracycline for which drug resistance has also spread, was extremely effectively loaded onto a rod-like DNA origami that improved the drug’s absorption and retention in leukemia cells. By comparing similar quantities of free daunorubicin and daunorubicin loaded onto DNA, it was discovered that the DNA origami increased the amount of daunorubicin delivered intracellularly, inhibited drug efflux, and hence prevented efflux-pump-mediated drug resistance [33]. When used with medicines, DNA origami structures have shown synergistic effects that include functioning as a regulated carrier for drug molecules, facilitating the drug’s efficient absorption, and strengthening the drug’s resistance to removal from the cell. F. Kong and colleagues. found that the DNA origami structure synergistically improved the reduction in cancer cells’ multidrug resistance and increased the therapeutic impact of the medication when the DOX-loaded rectangular DNA origami was placed into the center of an emulsion system [76]. Since DNA origami is bigger than the minuscule molecular medicines, intercalating it with DOX or daunorubicin has been shown to increase the amount of anti-tumor medication internalized and to inhibit drug efflux, hence overcoming efflux-pump-mediated drug resistance.

Many targeting ligands, as previously noted, can be linked to the DNA origami to enhance cellular absorption, drug transport, and administration. Triangle DNA origami is readily modified to integrate aptamers, a family of nucleic acids that may detect membrane receptors, trigger aptamer-receptor mediated endocytosis in HeLa cells, and dramatically increase the effectiveness of DOX administration [77]. It has been demonstrated that the effectiveness of cellular absorption is influenced by carefully adjusting the density and orientation of aptamers in DNA origami constructions [60]. Small molecules and proteins are examples of other targeted ligands that have been used for this purpose. The iron transport protein transferrin was incorporated onto the edge of DNA origami based on protein-receptor-mediated endocytosis. As a consequence, in KB-3-1 cell lines, the internalization efficiency of those DNA origamis showed an increase of up to 22-fold [78]. As a pre-clinical method to treat prostate cancer, Z. Ge et al. included a small molecule-targeted ligand named 2-[3-(1,3-dicarboxy propyl)-ureido] pentanedioic acid into DNA origami. Prostate-specific membrane antigen positive (PSMA+) tumor cells showed the best internalization efficiency for ligand-modified DNA origami because of the precise binding of targeted ligands and antigens [79]. Overall, the targeting effectiveness and particular binding efficiency of DNA origami structures are directly caused by the interplay between ligand modifications and membrane receptors (Figure 4C).

Less research has been conducted on the distribution of therapeutically carried DNA origami in vivo than has been performed in vitro; however, there has been some substantial in vivo DNA origami research performed. One of the earliest in vivo studies of DNA origami/ DOX dispersion was given by Q. Zhang et al. This research used triangle-shaped DNA origami nanostructures to provide remarkable therapeutic outcomes in vivo without systemic toxicity and a high passive tumor targeting accumulation of DOX in cancer regions [27]. In addition to DNA intercalator medicines, protein drugs have also been incorporated into DNA nanostructures for in vivo testing. For the purpose of transporting a protein for therapeutic benefits, an autonomous DNA nanorobot was created. An external DNA aptamer on this robot induced the inside cavity to open up and expose conjugated thrombin, which led to coagulation, necrosis, and the in vivo suppression of tumor formation [24,25] when it came to contact with the target nucleolin (Figure 4B). Nucleic acid medicines are an important class of treatments that have been combined with DNA nanostructures for the treatment of malignant cells. By integrating antisense RNA into DNA nanostructures, nucleic acid self-assembly is a feasible method for producing nucleic acid therapies resistant to enzyme breakdown. The first rectangular and tubular DNA origami structure was created, transfected into tumor cells for in vivo gene therapy, and silenced the mRNA of BCL-2 using short interfering RNA, according to M. A. Rahman et al. The results showed that 70% of BCL-2 mRNA was silenced, and up to 90% of BCL-2 was knocked out [80]. To overcome the limitations of intercalator medicines, Q. Pan et al. created rectangular DNA origami constructs containing anti-sense oligonucleotides of mRNAs that translate P-glycoprotein and B-cell lymphoma 2 protein. In the HeLa/ADR and MCF-7/ADR cell lines, these structures showed therapeutic actions that worked in concert to reverse drug resistance [62]. J. Liu et al. constructed nanostructures with identical properties and tested them in vivo even though RNA interference in vivo was not fully covered in this work. In the research, drug-resistant MCF-7R tumors were treated with DOX and a special triangle-shaped DNA origami nanostructure. The DNA nanodevice had low systemic toxicity, inhibited the growth of tumors, and might be used in tumor gene therapy [69]. In an incremental study, the same scientists used the same triangular DNA origami to transport DOX and nucleic acid drugs to fight drug-resistant tumor cells. These nucleic acid drugs use two linear RNA transcription templates to target the protein P-glycoprotein and the anti-apoptotic protein survivin. Three complementary therapeutic strategies were used by the nanocarriers to effectively penetrate MCF-7R cells and limit the growth of tumors in vivo [81]. The DNA origami nanoplatform was used to load and distribute short hairpin RNA (shRNA) transcription templates encoding the multidrug resistance genes P-glycoprotein and survivin [82]. Several treatment agents are typically necessary in concert with nucleic acid drugs to achieve the most effective suppression of tumors in vivo [59,83]. The absorption and penetration of DNA-origami-based delivery in cell and cell spheroid tissue models (CSTMs) are investigated in a study to see whether changes in internal structure might be a role in effectiveness. Two structures are constructed with basically comparable characteristics in terms of geometry and molecular weight, but with differing interior designs—either compact, or an open wireframe design, lattice-based origami. Wireframe rods can penetrate deeper than close-packed rods in CSTMs. Furthermore, DOX-loaded wireframe rods exhibit increased cytotoxicity in CSTMs. The variations in permeability, local deformability, local material density, and structural mechanics to cell receptors between these two DNA origami design paradigms help explain these findings. The fundamental cause for the variation in penetration dynamic is thought to be the differences in interaction with scavenger receptors, wherein lattice-based structures tend to be internalized to a greater extent than polygonal nanostructures that are identical in shape and dimension. It is therefore claimed that the selection of a structural design approach is a critical parameter for the implementation of DNA origami in the administration of drugs [84].

To explore their cell transport activities, DNA origami nanostructure carriers are frequently tagged with fluorescent dyes. Because such fluorescent dyes are observable under a microscope, they are perfect model molecules for drug carriers to transport into cells (Figure 4D). It has been demonstrated that DNA nanotubes engineered with Cy3 molecules may invade human tumor cells. DNA nanotubes might be internalized by tumor cells with the assistance of folate, triggering the development of folate receptors. Ding and colleagues have disclosed a label-free fluorescent approach for studying the dispersion and durability of DNA origami nanostructures in living cells. A dye substance called carbazole-based biscyanine might link to a DNA duplex and generate high fluorescence in a compact DNA structure. Yet, when the compact DNA structure was disturbed, the intensity of its fluorescence decreased. DNA origami structures were used to transport carbazole-based biscyanine into cells, and it has been found that this nanocarrier could withstand degradation for up to 60 h in cells, providing strong examples for regulating cargo release [85].

## 6. Challenges and Future Perspectives of DNA Origami Nanostructures as Delivery System

**Figure 5:**
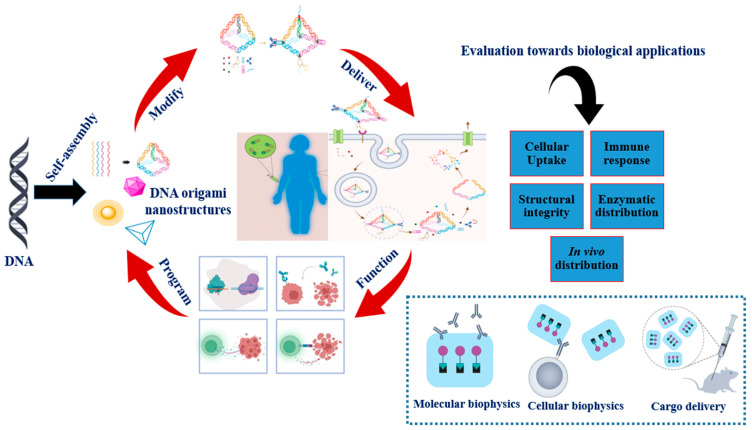
Current opportunities and challenges of DNA origami nanostructures for controlled therapeutic drug delivery.

Molecular self-assembly is widely employed in the areas of effective catalyst materials, biosensors, medicinal biomaterials, and molecular devices. DNA is a biomacromolecule made up of deoxyribonucleotide units, which serves as the transmitter of genetic data. DNA nanotechnology transcends DNA’s initial features as a molecule that stores and transfers genetic information from its biological environment by utilizing its distinctive base pairing and innate biocompatibility to construct structurally defined supramolecular frameworks. As DNA technology advances, the assembly process of DNA nanostructures is no longer confined to DNA hybridization but includes other biological interactions [86]. Below, we summarize the major challenges that need to be addressed on a general basis before DNA origami can be applied broadly (Figure 5).

### 6.1. Challenges

[A] Cost: One of the most difficult obstacles standing in the way of the practical uses of DNA origami as a medication delivery system is cost. At a synthesis scale of roughly 10 nmol, staple strands of a 7000 bp origami structure are commonly available for purchase for several hundred dollars. The true cost of individual DNA origami design would be significantly greater if other expenses, such as scaffold DNA, oligonucleotide functionalization, and origami purification, were taken into consideration. Therefore, it is vitally necessary to develop cost-effective scaffold and staple DNA synthesis techniques. Given that basic DNA tile structures have previously been generated in vivo, it may be possible to resolve this problem by producing DNA strands or perhaps whole origami structures in vivo [87].

[B] In vitro and in vivo stability: Another problem that has to be solved is the stability of DNA origami in vitro and in vivo. High quantities of cationic ions (such as Mg^2+^ and Na^+^) are necessary to neutralize the negative charge of the DNA backbone and maintain DNA origami structures because of the extraordinarily dense packing of DNA duplexes in these nanostructures. The amounts of cationic ions in typical physiological solutions (such PBS and medium) are insufficient to stabilize DNA origami constructs. The stability of DNA origami in fluids that simulate physiological circumstances has been tested in several experiments. Less dense things, such as wire-frame origami creations, have been discovered to be more stable in cation-depleted fluids [13,88,89].

[C] Immune response: Exogenous DNA insertion carries a number of risks, including long-term integration into the genome, induction of a strong immune response, and sequence-specific interference with mRNAs or microRNAs that results in undesired gene regulation. One possible answer to these issues is the chemical alteration of the fundamental DNA strands, such as the insertion of modified phosphoramidites or post-synthetic modification to make them physiologically inactive [90].

[D] Design: Further simplified and automated design platforms need to be developed, especially for researchers outside the DNA nanotechnology field.

[E] Scale up: The size of discrete origami structures is typically constrained within 100 nm because of the length of the M13 scaffold DNA, and thus, alternative strategies need to be developed for size expansion.

[F] Chemical functionality: DNA is a relatively chemically inert biomolecule, and thus, facile methods for adding a wide variety of functionalities need further development. Current methods for introducing additional reactivity through the introduction of alternative nucleotides during synthesis or post-synthetic modifications provide a good starting point but can be cost prohibitive.

[G] Defects: DNA origami structures contain assembly defects, which can hinder heteroelement or therapeutic incorporation. Optimizing structural designs (e.g., crossover pattern and staple length) and assembly conditions (e.g., Mg^2+^ concentration and a thermal annealing protocol) can help to minimize structural defects. Straightforward methods should also be developed to allow a convenient examination of structural quality.

### 6.2. Future Perspectives

[A] Predictable and well-defined structure: It is generally known that object size and form can affect how cells internalize substances. Given the ease and flexibility with which DNA origami nanostructures of various sizes and forms may be designed and produced, this adaptability gives us a great chance to experiment with different structures as drug carriers, and there is potential to optimize a number of different factors for cellular uptake using the same uniform material [91].

[B] Stability: An important criterion is how stable DNA nanostructures are in a physiological environment. It has been demonstrated that in nuclease-containing circumstances, DNA origami are more stable than ssDNA and regular DNA duplexes. This stability may be caused by the fact that the DNA origami’s odd forms and structures have physical complexity that make it difficult for nucleases to access and use them. It has been demonstrated that various DNA origami nanostructures may survive for 12 h in cell lysates at room temperature without deteriorating [43].

[C] Drug loading and release: The flexibility of DNA origami nanostructures’ drug loading and release properties makes them useful for designing the structural elements of nanocarriers. Unmethylated cytosine–phosphate–guanine (CpG) sequences have been employed as a model cargo and have been covalently attached to DNA nanocarriers in order to elicit an immunological response. A Fab fragment, AuNPs, and active enzymes have all been reported to be contained inside a DNA origami nanostructure’s hollow. The cargo and enzymes were able to be more stable, catalytically active, and resistant to protease digestion because of these DNA origami nanostructures, according to the data [51,67,92].

[D] Cellular internalization: It has been demonstrated that DNA origami nanostructures with larger sizes and stronger compactness enable more effective internalization than structures with smaller compactness or isolated ssDNA [67]. DNA origami nanostructures have been altered with targeted ligands, such as folate, cell-penetrating proteins, and transferrin, to increase the efficiency of cellular absorption [93]. Additionally, improved permeability and retention (EPR) effects were seen in DNA origami nanostructures. After an intravenous injection into tumor-bearing mice, the passive accumulation of DNA origami in the three distinct forms of triangle, rectangle, and tube was examined using QD labelling. It was discovered that 24 h after injection, the triangles accumulated at the tumor site at greater quantities than the tubular nanostructures [27].

[E] Therapeutic efficacy: The high loading, minimal cytotoxicity, perfect stability, and releasing capability of nanocarriers all contribute to high effectiveness in cancer therapy. Numerous studies have shown that DNA origami nanostructures improved anticancer functions and circumvented drug resistance. DOX-infused triangular and tubular DNA origami nanostructures, according to Jiang and colleagues, boosted the apoptosis of DOX-resistant breast cancer [76]. It has been suggested that DNA nanocarriers can lessen the negative effects of chemotherapy. When compared to mice in the free-drug group, animals treated with DOX-containing DNA triangles efficiently reduced tumor growth while causing minimal weight loss, demonstrating that these DNA nanocarriers were less harmful than free-drug mice [27].

[F] Photodynamic therapy: In photodynamic therapy (PDT), cancer cells are killed by combining light with photosensitizers. There are several photosensitizers for PDT, including silicon phthalocyanine Pc 4, aminolevulinic acid, and porphyrins. Some medicines, however, have drawbacks such as slow absorption, quick clearance, and poor solubility, which therefore, lead to insufficient therapeutic effectiveness. Additionally, DNA origami nanostructures have been applied in PDT as nanocarriers of photosensitizers [94].

[G] Further investigations: To completely understand the stability issue with DNA origami constructs, more research is required. Although the effective cell entry of DNA origami structures has been experimentally confirmed, the precise endocytosis process has not yet been identified through rigorous mechanistic research. Another difficult obstacle is that every study has indicated that DNA origami constructs eventually make their way to lysosomes for digestion. DNA origami vehicles may be required to escape from the lysosome in order to facilitate an effective cargo release into the cytosol. Potential tactics include conjugating functional molecules onto DNA origami to promote lysosomal escape or utilizing targeted ligands to start absorption via a non-lysosomal route. Before any clinical drug-delivery applications, a deeper comprehension of the pharmacokinetics and pharmacodynamics of DNA origami constructs in vivo is also required [95].

## 7. Conclusions

Because of its improved drug targeting and lower drug toxicity, nanomedicine has grown at an exponential rate. It makes use of interactions in which nanotechnological materials and biological systems interface with one another to improve delivery performance.

The diversified DNA nanostructures have evolved into some of the most prominent self-assembly systems with a lengthy and notable history. Since it was first discovered, DNA origami has made incredible progress toward a variety of biological applications. This paper covers several approaches currently utilized for the construction of DNA origami nanostructures. The use of these DNA nanostructures with well-defined parameters for accurate control in the delivery of drug and gene therapy is also explored. Several possible applications of DNA origami have been presented, covering areas from nanomaterials to drug delivery, and they have demonstrated potential as strategies of interacting with cells. The cellular transport of DNA nanostructures is an emerging field of interest, which we cover in this review, concentrating largely on DNA origami structures. We pointed out several drugs that can be encapsulated with DNA origami and we concluded the prospectives and challenges of DNA origami from our point of view. While DNA-origami-based nanotechnology has great potential for precise nanomedicine, it is still in its early stages. Prior to clinical translation, certain critical obstacles must be addressed: (I) Unclear operating mechanism. Although DNA nanostructures have been explored for drug delivery, more research into the mechanics of transfection is desperately required since the real mechanism of uptake and how crucial parameters, such as size and shape, impact uptake are still unknown; (II) Further testing of safety profile. DNA, being a naturally biocompatible and biodegradable polymer, performs quite well in some types of cells and in mice. In these preliminary trials, there was no antibody reaction against DNA nanostructures. Nevertheless, considering the complexities of the human body, the impact of particle physicochemical characteristics on renal systems, and the doubtful but potentially harmful genome recombination, more research on DNA origami nanostructures in different types of organs is required before they can be used in clinical settings. The practical implementation of DNA origami nanostructures in vivo would be driven by additional mechanistic research on the fate of DNA nanostructures in vivo and the development of effective ways to reduce interruption from the physiological environment. Chemically altered DNA origami with many customizable sites has the ability to accurately co-load and effectively co-deliver a variety of therapeutic drugs via a protective barrier to improve nuclease resistance. We anticipate that in the near future, such chemically altered DNA origami nanostructures will be extensively utilized in the construction of drug carriers and we also be applicable to pre-clinical and clinical investigations. This type of strategically designed drug delivery method is important in reducing the systemic toxicity found in conventional administration and improving medication pharmacodynamics. This study encourages the advancement of innovative DNA-origami-based targeted drug delivery for the efficient and appropriate co-delivery of encapsulated cargoes in anti-cancer therapeutic applications, and also paving the way for the future medical applications of DNA origami nanostructures as prospective delivery platforms for tumor-targeted therapies. We believe that with further advancement in drug delivery systems and with the resolution of scalability difficulties, DNA-origami-based nanotechnology can bring a new notion into carrier systems and provide beneficial clinical results. Recent discoveries in the design and implementation of DNA origami nanostructures demonstrate that there is substantial potential for improvement, which should lead to significant applications for these nanostructures in material sciences and healthcare. These findings may contribute to the development and optimization of DNA origami nanostructures as drug carrier nanoplatforms for biomedical applications. This topic will undoubtedly attract multidisciplinary research efforts among chemists, biologists, doctors, and bioengineers, and interesting new discoveries will emerge.

## Figures and Tables

**Table 1:** List of selected functionalized DNA origami nanostructures used in the recent literature with their diameter, length, therapeutic agent, cell lines, targeted model, and targeted sites.

Shapes of DNA Origami Nanostructures	Diameter	Length	Therapeutic Agent/Drug Loaded	Cell Lines	Targeting Ligands	Targeted Sites or Triggered Conditions	Target Model	Ref.
Triangle-shaped DNA origami	12.5 nm.	67 nm.	BMEPC	MCF-7	-------	Photodynamic therapy (PDT)	In vitro	[58]
Triangle-shaped DNA origami	12.5 nm.	67 nm.	Gold nanorods	4T1-fLuc	-------	Photodynamic therapy	In vitro	[58]
Triangle-shaped DNA origami	12.5 nm.	67 nm.	DOX,Gold nanorods.	MCF-7/ADR	-------	Photothermal therapy/MUC1	Orthotopic transplantation	[53]
Triangle-shaped DNA origami	12.5 nm.	67 nm.	Therapeutic gene p53, DOX	MCF-7R, MCF-7	MUC1 aptamers	MUC1/genes	Subcutaneous xenograft	[59]
Triangle-shaped DNA origami	12.5 nm.	67 nm.	hairpin RNA, DOX	MCF-7R, MCF-7	MUC1 aptamers	MUC1/genes	In vitro	[52]
Triangle-shaped DNA origami	12.5 nm.	67 nm.	DOX	HeLa	Sgc8 aptamers	PTK7	In vitro	[60]
Tube-shaped DNA origami	2.25 nm.	0.34 nm.	DOX	MDA-MB-231, MDA-MB-468, MCF-7	-------	-------	In vitro	[61]
Tube-shaped DNA origami	2.25 nm.	0.34 nm.	DOX	LNCaP (PSMA+), PC-3	DUPA	PMSA	In vitro	[24]
Rectangle-shaped DNA origami	70 nm.	2 nm.	Antisense oligonucleotides	HeLa/ADR, MCF-7/ADR	MUC1 aptamers	mRNA of B-cell protein and P-glycoprotein	In vitro	[62]
Rectangle-shaped DNA origami	70 nm.	2 nm.	5-fluoro-2′ -deoxyuridine	HTB-38, HCC-2998	-------	-------	In vitro	[27]
Tube-, triangle-shaped DNA origami	Tube—2.25 nm. Triangle—12.5 nm.	Tube—0.34 nm. Triangle—67 nm.	Gold nanorods	MCF-7	-------	Photothermal therapy	Subcutaneous xenograft	[63]
Tube-, triangle-shaped DNA origami	Tube—2.25 nm. Triangle—12.5 nm.	Tube—0.34 nm. Triangle—67 nm.	DOX	MCF-7R	-------	-------	In vitro	[34]
Rectangle-, tube-shaped DNA origami	Rectangle—70 nm. Tube—2.25 nm.	Rectangle—2 nm. Tube—0.34 nm.	Thrombin	HUVECs, MDA-MB-231, SK-OV3, B16-F10, bEnd.3	AS1411 aptamers	Nucleolin	In vitro	[62]
Rectangle-, tube-shaped DNA origami	Rectangle- 70 nm. Tube—2.25 nm.	Rectangle- 2 nm. Tube—0.34 nm.	siRNA	DMS53, H1299	-------	mRNA of BCL-2	In vitro	[34]
Tube-, rectangle-, triangle-shaped DNA origami	Tube—2.25 nm. Rectangle—70 nm. Triangle—12.5 nm.	Tube—0.34 nm. Rectangle—2 nm. Triangle—67 nm.	DOX	MDA-MB-231	-------	-------	In vitro	[53]

## Data Availability

Not applicable.
